# Protective effect of Growth Hormone-Releasing Hormone agonist in bacterial toxin-induced pulmonary barrier dysfunction

**DOI:** 10.3389/fphys.2014.00259

**Published:** 2014-07-15

**Authors:** Istvan Czikora, Supriya Sridhar, Boris Gorshkov, Irina B. Alieva, Anita Kasa, Joyce Gonzales, Olena Potapenko, Nagavedi S. Umapathy, Helena Pillich, Ferenc G. Rick, Norman L. Block, Alexander D. Verin, Trinad Chakraborty, Michael A. Matthay, Andrew V. Schally, Rudolf Lucas

**Affiliations:** ^1^Department of Pharmacology and Toxicology, Vascular Biology Center, Georgia Regents UniversityAugusta, GA, USA; ^2^Department of Electron Microscopy, A.N. Belozorksy Institute, Moscow State UniversityMoscow, Russia; ^3^Department of Medicine, Division of Pulmonary Medicine, Medical College of Georgia, Georgia Regents UniversityAugusta, GA, USA; ^4^Department of Medicine, Institute of Medical Microbiology, Justus-Liebig University GiessenGiessen, Germany; ^5^Endocrine, Polypeptide and Cancer Institute, Veterans Affairs Medical CenterMiami, FL, USA; ^6^Department of Urology, Herbert Wertheim College of Medicine, Florida International UniversityMiami, FL, USA; ^7^Department of Pathology, Miller School of Medicine, University of MiamiMiami, FL, USA; ^8^Department of Medicine and Anesthesia, Cardiovascular Research Institute, University of California San FranciscoSan Francisco, CA, USA; ^9^Department of Medicine, Miller School of Medicine, University of MiamiMiami, FL, USA; ^10^Sylvester Comprehensive Cancer Center, Miller School of Medicine, University of MiamiMiami, FL, USA; ^11^Department of Pharmacology and Toxicology, Medical College of Georgia, Georgia Regents UniversityAugusta, GA, USA

**Keywords:** capillary leak, pneumolysin, lipopolysaccharide, growth hormone-releasing hormone, protein kinase A, protein kinase C

## Abstract

**Rationale:** Antibiotic treatment of patients infected with G^−^ or G^+^ bacteria promotes release of the toxins lipopolysaccharide (LPS) and pneumolysin (PLY) in their lungs. Growth Hormone-releasing Hormone (GHRH) agonist JI-34 protects human lung microvascular endothelial cells (HL-MVEC), expressing splice variant 1 (SV-1) of the receptor, from PLY-induced barrier dysfunction. We investigated whether JI-34 also blunts LPS-induced hyperpermeability. Since GHRH receptor (GHRH-R) signaling can potentially stimulate both cAMP-dependent barrier-protective pathways as well as barrier-disruptive protein kinase C pathways, we studied their interaction in GHRH agonist-treated HL-MVEC, in the presence of PLY, by means of siRNA-mediated protein kinase A (PKA) depletion.

**Methods:** Barrier function measurements were done in HL-MVEC monolayers using Electrical Cell substrate Impedance Sensing (ECIS) and VE-cadherin expression by Western blotting. Capillary leak was assessed by Evans Blue dye (EBD) incorporation. Cytokine generation in broncho-alveolar lavage fluid (BALF) was measured by multiplex analysis. PKA and PKC-α activity were assessed by Western blotting.

**Results:** GHRH agonist JI-34 significantly blunts LPS-induced barrier dysfunction, at least in part by preserving VE-cadherin expression, while not affecting inflammation. In addition to activating PKA, GHRH agonist also increases PKC-α activity in PLY-treated HL-MVEC. Treatment with PLY significantly decreases resistance in control siRNA-treated HL-MVEC, but does so even more in PKA-depleted monolayers. Pretreatment with GHRH agonist blunts PLY-induced permeability in control siRNA-treated HL-MVEC, but fails to improve barrier function in PKA-depleted PLY-treated monolayers.

**Conclusions:** GHRH signaling in HL-MVEC protects from both LPS and PLY-mediated endothelial barrier dysfunction and concurrently induces a barrier-protective PKA-mediated and a barrier-disruptive PKC-α-induced pathway in the presence of PLY, the former of which dominates the latter.

## Introduction

Of the approximately 9 million children aged less than 5 years that die each year worldwide, 1.6 million succumb from pneumonia (Rudan et al., 2008). Indeed, pneumonia kills more children world-wide than any other single illness. In the US more than 5 million cases of community-acquired pneumonia (CAP) are diagnosed annually, mainly in the elderly population, with 7% of them fatal (Waterer et al., 2011). Despite the widespread use of antibiotics, mortality during the first several days of pneumonia has not decreased appreciably over the past 75 years (Evans and Gaisford, 1938; Waterer et al., 2011). Even though the lungs are essentially free of bacteria following antibiotic treatment, many patients still die from complications, the most important one of which is pulmonary permeability edema. One of the main causes of pneumonia-associated permeability edema in patients treated with antibiotics is the massive release of toxins, including pneumolysin (PLY), a virulence factor from G^+^ pneumococci and lipopolysaccharide (LPS), an endotoxin from G^−^ bacteria (Anderson et al., 2007), following bacterial lysis. Although inducing very different pathways leading to endothelial dysfunction, both toxins are important mediators of pneumonia-associated capillary leak, which is an important problem contributing to permeability edema.

Endothelial and epithelial barrier functions are regulated by a tight balance between opposing centripetal and centrifugal intracellular forces, provided by the contractile machinery and the elements opposing contraction, respectively. The elements opposing barrier dysfunction include tethering complexes, involved in cell-cell and cell-matrix contacts, and systems that impart cell rigidity and prevent cellular collapse, such as actin filaments, microtubules, and intermediate filaments (Dudek and Garcia, 2001; Lucas et al., 2009).

The 53 kDa cytoplasmic cholesterol-binding and pore-forming cytolysin, PLY, is a major pneumococcal virulence factor, and exerts its barrier-disruptive effects mainly through its direct activities on myosin light chains (MLC) and on microtubules (Rubins et al., 1996; Witzenrath et al., 2006; Lucas et al., 2012a,b, 2013), rather than through stimulation of leukocyte infiltration and cytokine generation (Maus et al., 2004). By contrast, the G^−^ endotoxin, LPS, upon stimulation of TLR4 (Beutler and Moresco, 2008), directly affects endothelial barrier function, by means of a mechanism involving RhoA GTPase activation-dependent downregulation of expression of the adherens junction protein, VE-cadherin (Schlegel et al., 2009; Rafikov et al., 2014). LPS-induced endothelial barrier dysfunction can be blunted by cAMP-dependent activation of protein kinase A (PKA) (Csortos et al., 2008; Bogatcheva et al., 2009; Umapathy et al., 2010). In contrast to PLY, LPS also indirectly promotes hyperpermeability, by means of potently activating leukocyte infiltration, inducing oxidative and nitrosative stress. In addition, increased generation of pro-inflammatory cytokines (Umapathy et al., 2012; Aggarwal et al., 2014; Gonzales et al., 2014), such as TNF, dramatically affects microtubule arrangement in the lung endothelium (Petrache et al., 2003). Currently, no standard treatment exists to counteract LPS- or PLY-mediated permeability edema. As such, the search for novel protective substances which can blunt endothelial barrier dysfunction induced by these bacterial toxins is of high clinical importance.

Generated in the posterior hypothalamus, the nominative function of the Growth Hormone Releasing Hormone (GHRH) is the stimulation of Growth Hormone production by the pituitary, which expresses full length GHRH receptors (GHRH-R) (Mayo et al., 1995; Sherwood et al., 2000). We have previously shown that mRNA for the ligand GHRH and its bioactive receptor splice variant, SV1, are also expressed in peripheral tissues, such as in lung microvascular endothelial cells, pancreatic islets, and cardiomyocytes (Rekasi et al., 2000; Kanashiro-Takeuchi et al., 2010; Kiaris et al., 2011; Ludwig et al., 2012).

The 29 N-terminal amino acid residues of the GHRH sequence possess full biological activity and thus constitute the core peptide for the development of agonists of GHRH, such as JI-34, JI-36, and JI-38, which are up to 80 times more potent than GHRH (Izdebski et al., 1995; Cai et al., 2014). JI-34 was shown to protect from PLY-induced barrier dysfunction *in vitro* and *in vivo*, in a cAMP-dependent manner (Lucas et al., 2012a). However, the outcome of GHRH agonist treatment on LPS-induced hyperpermeability in capillary endothelial cells *in vitro* and *in vivo* has not been investigated.

Ligand or agonist binding to the GHRH-R changes receptor conformation and activates the closely associated heterotrimeric Gs-protein. Upon this activation, the dissociated Gαs subunit directly stimulates adenylate cyclase and intracellular cAMP generation, which in turn activates PKA (Moretti et al., 2002). However, GHRH belongs to the glucagon/secretin superfamily that has been demonstrated to activate receptors coupled with multiple heterotrimeric G proteins, particularly Gs and Gq (Moretti et al., 2002; Lania et al., 2003). Therefore, in addition to cAMP accumulation and PKA activation via Gs, these receptors may also potentially induce a rise in intracellular Ca^2+^ and PKC activation via Gq (Lania et al., 2003).

The main aims of this study were to investigate whether apart from PLY, GHRH agonists can also protect from LPS-induced barrier dysfunction. We also investigated whether GHRH agonists are able to activate both PKA-mediated barrier-protective and PKC-mediated barrier-disruptive pathways in human lung microvascular endothelial cells (HL-MVEC) and how these pathways interact.

## Materials and methods

### Cells

Human lung microvascular endothelial cells (HL-MVEC) and human pulmonary artery endothelial cells (HPAEC) (Lonza, Walkersville, MD, USA) were grown in complete EBM-2 medium (Lonza, Walkersville, MD, USA) and used up to passage six. Experiments with PLY were performed in serum-free medium, whereas experiments with LPS were performed in medium containing 5% FBS.

### Mice

Eight to ten weeks old male C57BL6 mice, weighing 19–21 g were obtained from Harlan and were kept at the animal facilities at Georgia Regents University. All animal studies conformed to National Institutes of Health guidelines. The experimental procedure was approved by the Georgia Regents University Institutional Animal Care and Use Committee.

### PLY purification

PLY was purified from a recombinant *Listeria innocua* 6a strain expressing LPS-free PLY in the laboratory of T.C. The batch of PLY used in this study had a specific activity of 1.25 × 10^7^ hemolytic units/mg.

### Biochemicals

Rabbit polyclonal anti–human vascular endothelial (VE)-cadherin antibodies, anti-human PKA, anti-human phospho-PKA, anti-human PKA substrate, anti-human PKC substrate, and anti-human PKC-α were from Cell Signaling Technology (Danvers, MA, USA). Rabbit anti-human phospho PKC-α(Ser 657) was from Santa Cruz Biotechnology (Dallas, TX, USA). Secondary goat anti-rabbit Texas Red-conjugated antibody and goat anti-rabbit secondary antibodies conjugated to Horse Radish Peroxidase (HRP) were from Sigma-Aldrich (St Louis, MO). LPS *E. coli* 0111:B4 was from Sigma (St Louis, MO).

### Peptide analogs preparation

GHRH agonist, JI-34, was synthesized in the laboratory of A.V.S. and is 80 times more potent in stimulating the GHRH-R than GHRH (Izdebski et al., 1995). For preparation of the stock solution, the agonist was dissolved in DMSO.

### Depletion of PKAα catalytic subunit and PKC-α in HL-MVEC

HL-MVEC were treated with a pool of 3 target-specific 19–25 nt siRNAs designed to knock down either PKAα catalytic subunit or PKC-α gene expression. These were obtained from Santa Cruz Biotechnology, Inc. (Dallas, TX, USA). Also a non-specific, non-targeting siRNA was ordered from the same manufacturer. All siRNA's were received in lyophilized form. HL-MVEC were transfected at 70–80% confluence with 75 nM final concentration of siRNA using siPORT™ Amine transfection reagent (Ambion, Life Technologies, Grand Island, NY) and used for further experiments at 48 h post transfection.

### Western blotting procedure

Immediately after treatment, HL-MVEC were washed twice with ice-cold PBS and lysed with lysis buffer [20 mM Tris·HCl (pH 7.6), 0.5% Nonidet P-40, 250 mM NaCl, 3 mM EDTA, 3 mM EGTA, 1 mM DTT, and protease inhibitor mixture]. The clear supernatants after centrifugation were mixed with SDS sample buffer and boiled for 5 min. Protein extracts were separated on SDS/PAGE, transferred to a nitrocellulose membrane, incubated with primary antibodies, and then incubated with HRP-conjugated secondary antibody. The immunoreactive proteins were visualized with LumiGLO solution (Cell Signaling, Danvers, MA, USA) and were then exposed to X-ray film. The relative intensity of each protein band was quantified with ImageJ software (National Institutes of Health).

### Measurements of VE-cadherin expression zone width

Analysis of VE cadherin zone expression was performed on immunofluorescent images we prepared using primary rabbit polyclonal anti-human VE-cadherin antibodies (Cell signaling, Danvers, MA, USA) and secondary goat anti-rabbit Texas Red-conjugated antibody (Sigma-Aldrich, St Louis, MO, USA) in HPAECs, which are better suited for this type of measurement than pulmonary microvascular endothelial cells. Images were acquired using a N-SIM super-resolution system, assembled on a TiE inverted microscope (Nikon, Japan) with a 100× oil immersion lens (*NA* = 1.49) and an iXon-897 EMCCD-camera (Andor, Ireland, effective pixel size 60 nm). Original images were processed using ImageJ software (Gaussian filtration and background subtraction). Measurements of the width of VE-cadherin expression zone were produced using ImageJ software (function “Measure”). Statistical analysis was performed using Sigma Plot 7.1 (SPSS Science, Point Richmond, CA) and Excel. Sigma Plot 7.1 software was used for graphical data presentation.

### Measurement of transendothelial electrical resistance (TER)

TER in HL-MVEC monolayers [electrical cell-substrate impedance sensing (ECIS) system 1600R; Applied Biophysics, Troy, NY, USA] was measured as described previously (Lucas et al., 2012a,b).

### Assessment of capillary leak *in vivo*

Mice (*n* = 10 per group), pretreated i.v. or not at −24 h and at −1 h before toxin instillation with JI-34 (100 μ g/kg) were anesthetized with i.p. ketamine (150 mg/kg) and acetylpromazine (15 mg/kg). Subsequently the trachea was exposed and LPS (Sigma-Aldrich, *E. coli* 0111:B4, 0.65 mg/kg in saline) was instilled i.t. for 24 h in anesthetized mice via a 20-gauge catheter. An Evans Blue dye (EBD)/Albumin mixture (30 mg/kg in saline; (0.5% EBD conjugated to 4% BSA, Fraction V; Sigma-Aldrich, St Louis, MO) was injected into the tail vein of 6 mice per group, 2 h before mice were killed, in order to assess vascular leak. Lungs free of blood were weighed and snap-frozen in liquid nitrogen. The left lung was homogenized, incubated with formamide (18 h at +60°C), and centrifuged at 5000 × g for 30 min. The optical density of the supernatant was determined spectrophotometrically at 620–750 nm. Extravasated EBD concentration in the lungs was calculated by using a standard curve (μ g of EBD per g of wet lung tissue), as described previously (Lucas et al., 2012a,b). For pro-inflammatory mediator determination, collected bronchoalveolar lavage fluid (BALF) from another 4 mice per group was centrifuged (500 × g for 15 min at 4°C), supernatant was centrifuged again (5000 × g for 15 min at 4°C), and pure BALF was used to measure cytokine/chemokine/growth factor concentrations using the multiplex MCYTOMAG-70K assay (EMD Millipore, Billerica, MA, USA), according to the manufacturer's instructions.

### Statistical analysis

All experimental data are presented as mean ± SD. Control samples and those obtained upon various stimuli were compared by unpaired Student's *t*-test. For multiple group comparisons, One-Way ANOVA was used. *p* < 0.05 was considered statistically significant.

## Results

### GHRH agonist preserves VE-cadherin expression in LPS-treated human endothelial monolayers *in vitro*

**Figure 1:**
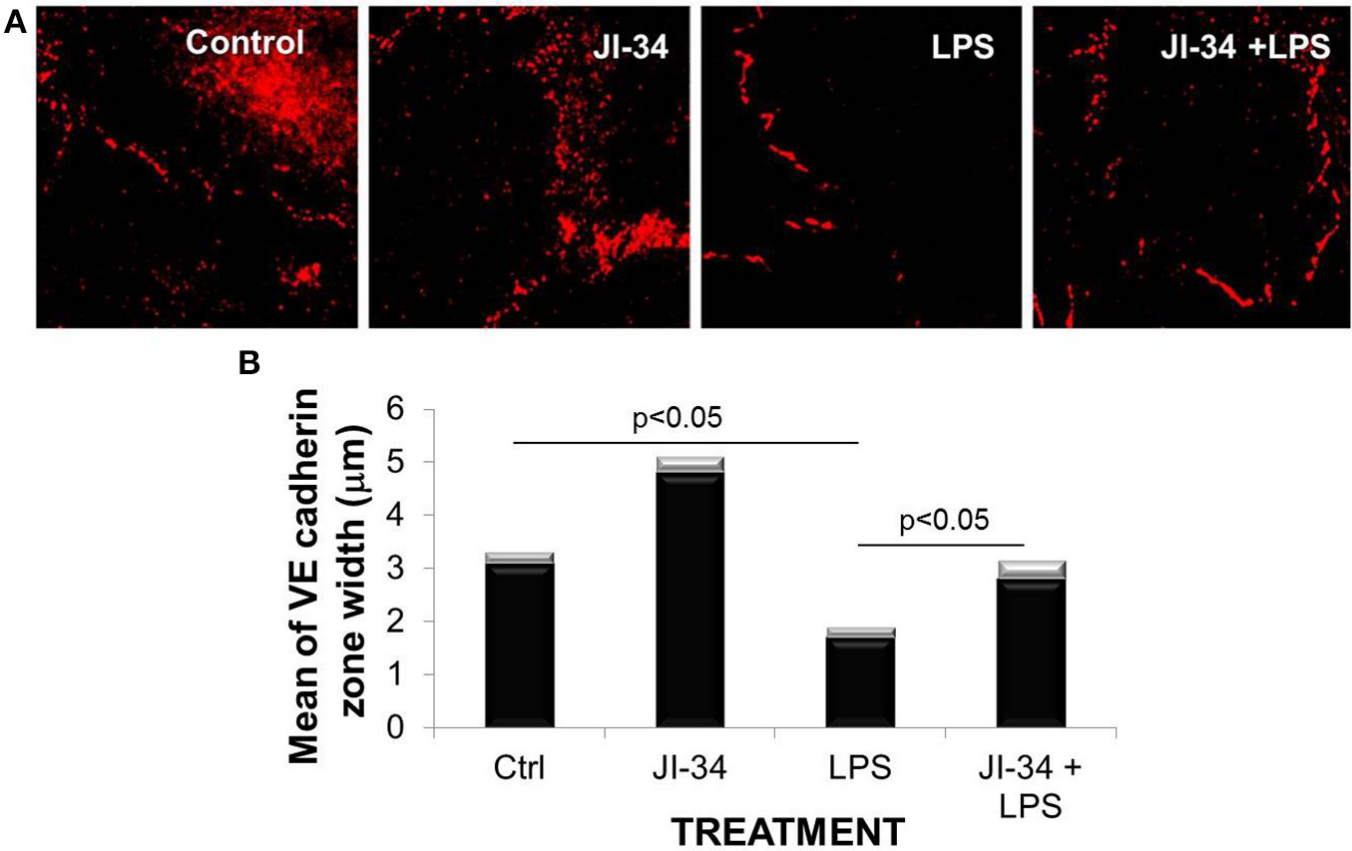
**Effect of LPS and JI-34 on VE-cadherin adherens junction of human pulmonary artery endothelial cells (HPAEC). (A)** Cells were pretreated with JI-34 (1 μ M, 15 min) or not, followed by LPS stimulation (500 ng/ml, 6 h) and stained with VE-cadherin antibodies for VE-cadherin adherens junction protein detection. **(B)** Measurements of VE-cadherin expression zone width were carried out on immunofluorescent images using ImageJ software. Mean of VE-cadherin zone width are presented as ± *SD* (gray bars) (*n* = 100 for each experimental treatment).

The main function of VE-cadherin, a crucial component of endothelial adherens junctions, is the stabilization of endothelial cell integrity. VE-cadherin expression was shown to be significantly reduced in the endothelium of all vessel types in the lungs of G^−^-sepsis-induced acute respiratory distress syndrome (ARDS) or in endothelial cells treated with LPS (Herwig et al., 2013). As shown in Figures 1A,B, treatment of monolayers of HPAECs for 6 h with LPS (500 ng/ml) leads to a significant reduction in VE-cadherin expression, measured as the mean of the VE-cadherin zone width (in μ m). Pretreatment of the monolayers for 15 min with the GHRH agonist, JI-34 (1 μ M), before LPS addition significantly blunted the effect of LPS and increased VE-cadherin expression even in control cells. In view of the crucial role of VE-cadherin expression for barrier function, these results indicate a barrier-protective effect of GHRH in LPS-treated endothelial cells *in vitro*. Since LPS exerts both direct and indirect (cytokine-mediated) deleterious actions on barrier function in the lung *in vivo*, in the subsequent study we investigated whether JI-34 can also blunt LPS-induced capillary leak in mice.

### JI-34 blunts LPS-induced pulmonary capillary leak *in vivo*

**Figure 2:**
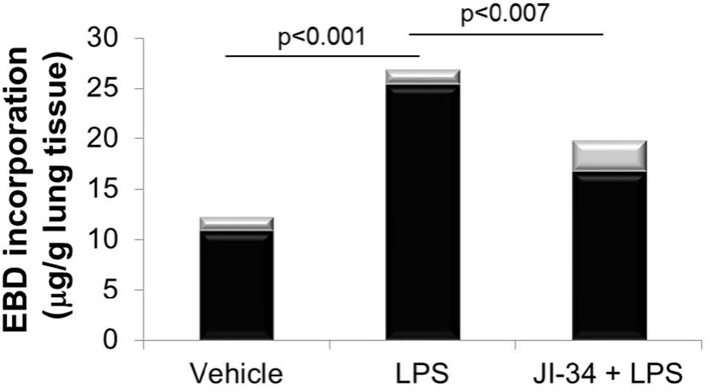
**Evans Blue Dye incorporation in lung tissue in 8–10 weeks old male C57BL6 mice (*n* = 6 per group) treated i.t. with 0.65 mg/kg LPS**. Mice were pretreated either with vehicle (DMSO) or with JI-34 (100 μ g/kg) at 24 and 1 h before receiving LPS. Data are given as mean ± *SD* (gray bars).

**Figure 3:**
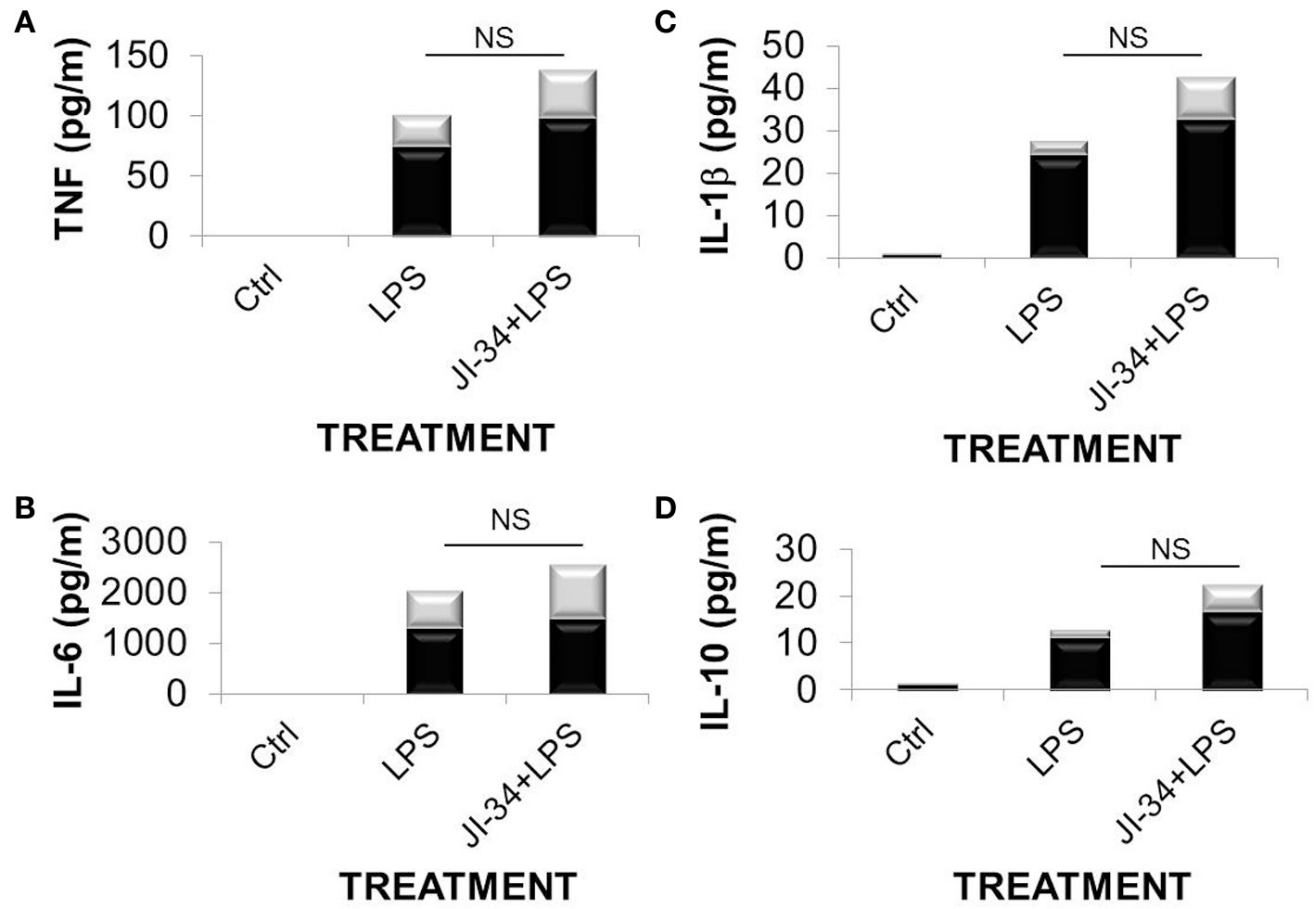
**Concentrations of (A) TNF; (B) IL-6; (C) IL-1 β; and (D) IL-10 in BALF from vehicle-treated, LPS-treated (0.65 mg/kg) or JI-34 (100 μ g/kg) + LPS treated mice (*n* = 4 per group) in pg/ml, as assessed using the Multiplex MCYTOMAG-70K assay (EMD Millipore, Billerica, MA, USA)**. Data are presented as mean ± *SD* (gray bars).

As shown in Figure 2, a combined i.v. pretreatment of 8 weeks old male C57BL6 mice (*n* = 6), with JI-34 (100 μ g/kg) at 24 and 1 h prior to i.t. LPS instillation (0.65 mg/kg) induced a significant protection from LPS-induced capillary leak, as compared to vehicle control and measured as EBD incorporation in the lung tissue. As depicted in Figure 3, JI-34 did not affect the concentration of the pro-inflammatory cytokines TNF, IL-1β, and IL-6 or of the anti-inflammatory cytokine IL-10 in BALF of LPS-treated animals, as measured by multiplex analysis.

**Figure 4:**
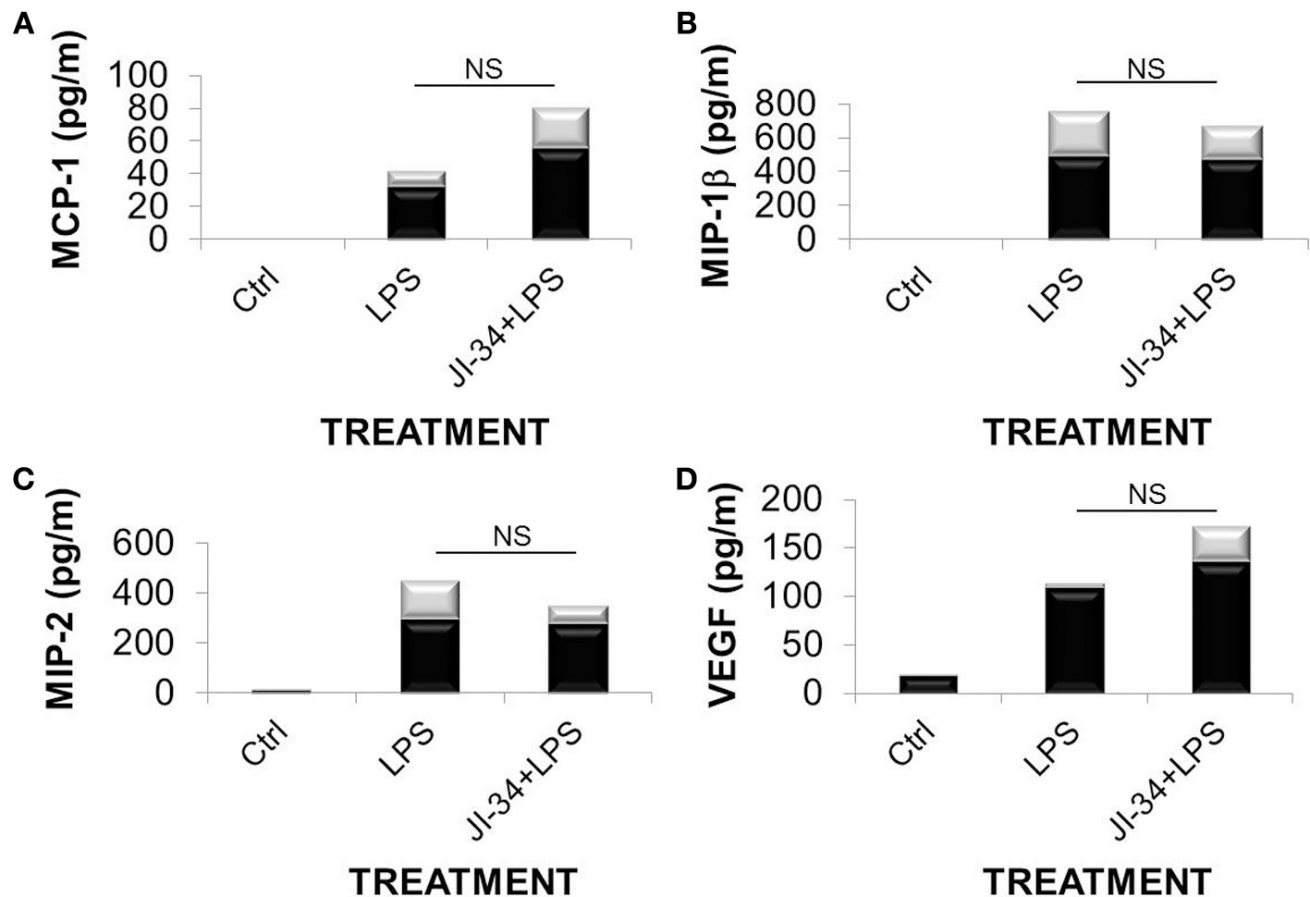
**Concentrations of the chemokines (A) MCP-1; (B) MIP-1 β; (C) MIP-2; and the growth factor (D)**. VEGF in BALF from vehicle-treated, LPS-treated or JI-34 + LPS treated mice (*n* = 4 per group) in pg/ml, as assessed using the Multiplex MCYTOMAG-70K assay (EMD Millipore, Billerica, MA, USA). Data are presented as mean ± *SD* (gray bars).

Moreover, neither the generation of the pro-inflammatory leukocyte-attracting chemokines MCP-1, MIP-1β, and MIP-2 nor of the permeability-increasing factor, VEGF, were affected by the GHRH agonist in LPS-instilled mice (Figure 4). These results suggest that the barrier-protective effect of JI-34 in lungs of LPS-treated mice results mainly from blunting direct, rather than indirect, leukocyte-mediated effects of LPS.

### Crucial role for protein kinase a in barrier-protective activity of GHRH agonists in human lung microvascular endothelial cells

In endothelial cells, cAMP potentiates VE-cadherin-mediated cell-cell contacts, causing enhanced endothelial barrier function (Dejana et al., 2008). The observed inhibitory activity of the GHRH agonist, JI-34, on both PLY-mediated (Lucas et al., 2012a) and LPS-induced VE-cadherin loss (Figures 1A,B), can thus, at least partially, be explained by its capacity to activate cAMP, upon activating the SV1 receptor, expressed in HL-MVEC. cAMP can however activate both PKA and Epac. PKA exerts its barrier-protective effects upon phosphorylating its substrates, including VASP, which was shown to be important in protecting endothelial barriers from LPS action (Bogatcheva et al., 2009). By contrast, Epac activates the barrier protective Rap1/Rac 1 cascade (Fukuhara et al., 2005).

**Figure 5:**
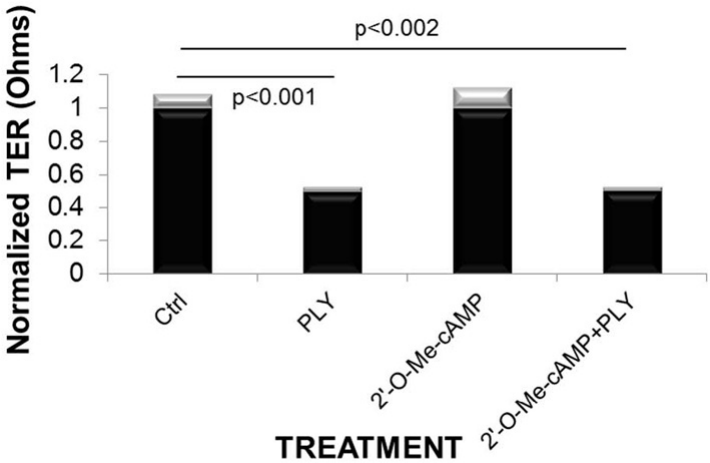
**The Epac agonist 2′-O-Me-cAMP (100 μ M) does not prevent PLY (30 ng/ml)-mediated hyperpermeability, measured as normalized transendothelial electric resistance (TER) in HL-MVEC (ECIS, *n* = 4, 6 h)**. Mean ± *SD* (gray bars).

Since agonists of both PKA and Epac can independently protect from LPS-induced hyperpermeability (Bogatcheva et al., 2009), we investigated whether specific activation of Epac, using the agonist 2′-O-Methyl-cAMP, can protect from PLY-mediated barrier dysfunction. As shown in Figure 5, 2′-O-Methyl-cAMP failed to restore barrier function in PLY-treated HL-MVEC, at a concentration of 100 μ M, which was previously demonstrated to protect from LPS-induced hyperpermeability in these cells (Bogatcheva et al., 2009). Since we have previously shown that cAMP protects from PLY-mediated barrier dysfunction in monolayers of HL-MVEC (Umapathy et al., 2010), this result suggests that the protective effect of the GHRH agonist JI-34 in PLY-mediated barrier dysfunction is dependent on the activation of PKA, rather than of Epac.

**Figure 6:**
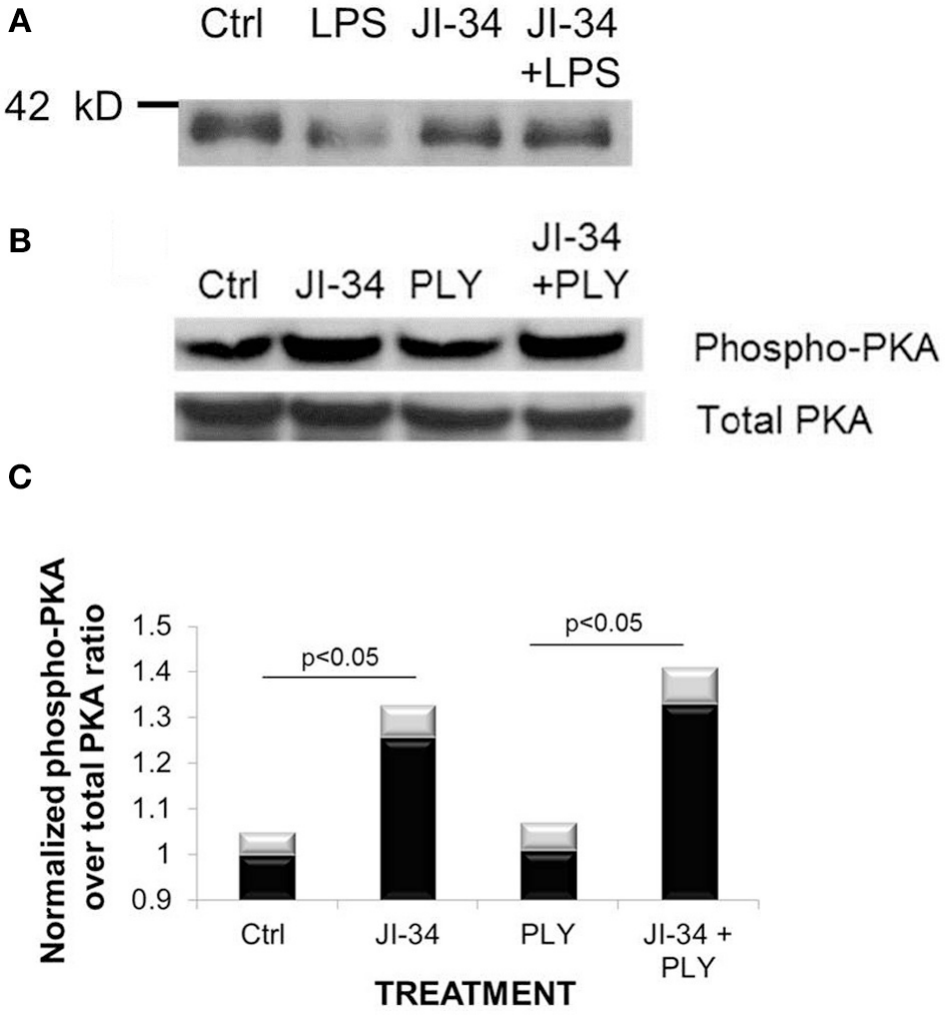
**Representative Western blots of the PKA-activating activity of a 30 min JI-34 preincubation (1 μ M) in ctrl, LPS (2 h, 200 ng/ml)- or PLY-(30 min, 30 ng/m) treated HL-MVEC. (A)** Expression of PKA protein substrates in LPS-treated or control cells, in the presence or absence of JI-34; **(B)** Phospho-PKA and total PKA protein expression in control and PLY-treated cells, in the presence or absence of JI-34. **(C)** Quantification of phospho-PKA over total PKA ratio (*n* = 3 per group, mean ± *SD*).

In order to substantiate this hypothesis, we investigated whether JI-34 can activate PKA in the absence or presence of LPS or PLY. As shown in Figure 6A, JI-34 (1 μ M) increases PKA activity, assessed as the expression of PKA-phosphorylated substrate protein in Western blotting in both resting and LPS-treated HL-MVEC monolayers. Further, as shown in Figures 6B,C, JI-34 (1 μ M) also activates PKA in PLY (30 ng/ml)-treated HL-MVEC, measured as the increased expression of phosphorylated over total PKA. Taken together, these results indicate that the activation of PKA, rather than of Epac, mediates the protective activity of JI-34 in both LPS- and PLY-mediated barrier dysfunction.

### Activation of SV-1 receptor in HL-MVEC can stimulate bacterial toxin-induced activation of PKC-α

**Figure 7:**
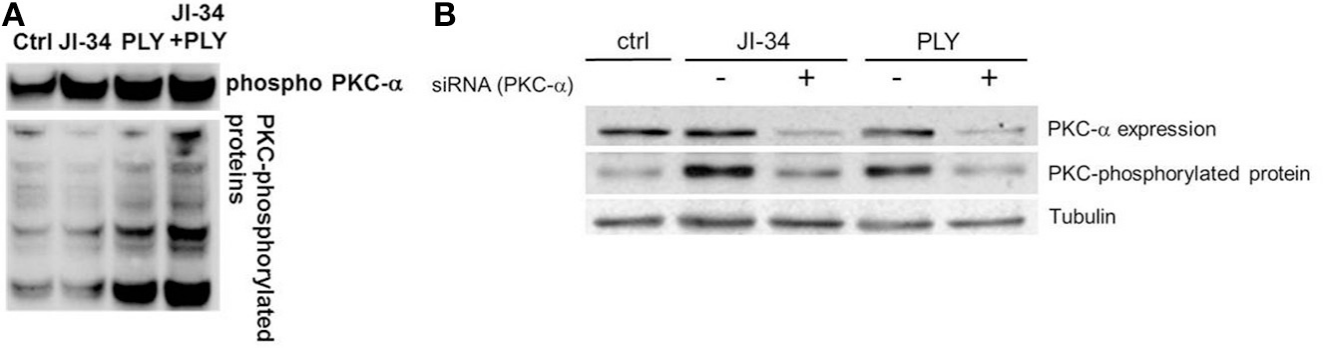
**(A)** Expression of PKC-phosphorylated protein substrates in ctrl, PLY (30 ng/ml), JI-34 (1 μ M), and JI-34 + PLY-treated HL-MVEC. Cells were pretreated for 30 min with JI-34 and were then incubated for 15 min with PLY. **(B)** Representative WB of the Influence of siRNA-mediated PKC-α gene depletion on PLY- and JI-34 induced total PKC activity in HL-MVEC.

**Figure 8:**
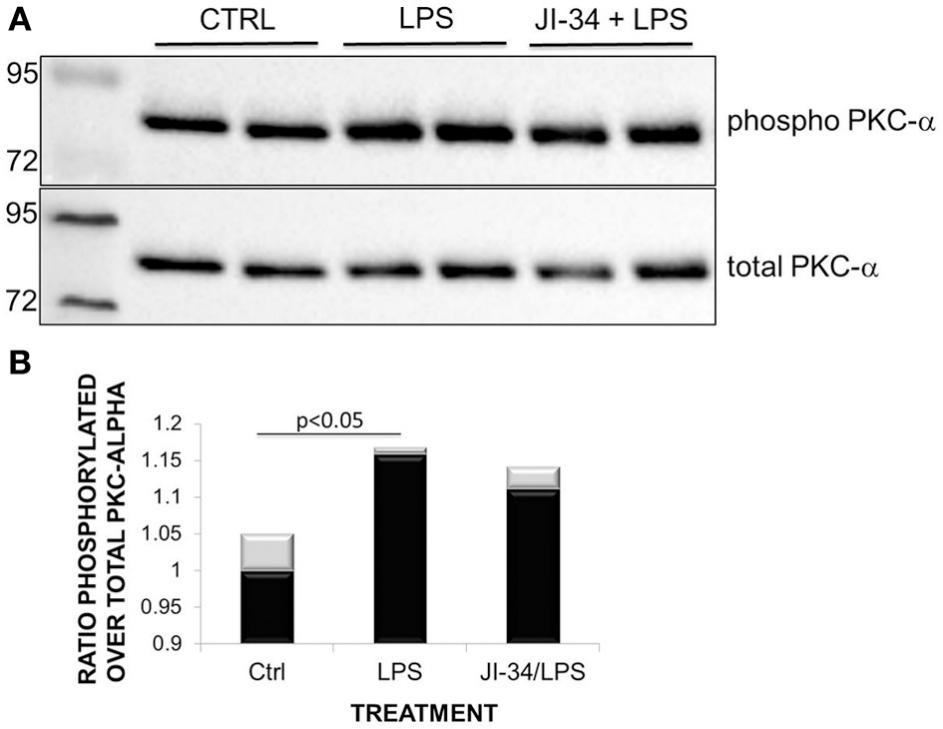
**Representative Western Blot demonstrating (A) phosphorylated PKC-α and total PKC-α activity in ctrl, LPS-treated (1 μ g/ml, 1 h) and JI-34(1 μ M, 15 min)/LPS-treated HL-MVEC**. **(B)** Phosphorylated over total PKC-α ratios. Mean + *SD* (gray bars) (*n* = 3 per group).

GHRH is a member of the glucagon/secretin superfamily, the members of which have the capacity to activate receptors coupled with multiple heterotrimeric Gq proteins, which have the capacity to increase intracellular Ca^2+^ (Mayo et al., 1995; Sherwood et al., 2000). The Gαq subunit, which was reported to be activated upon GHRH-R stimulation, has the capacity to activate PKC, after first stimulating phospholipase C (PLC) (Lania et al., 2003). In this study, we therefore investigated whether a 30 min incubation with JI-34 (1 μ M) of both resting cells and PLY-treated cells can increase PKC activity, assessed as the expression of PKC-phosphorylated protein substrate in Western Blotting. As shown in Figure 7A, a 30 min pretreatment of HL-MVEC monolayers with JI-34 (1 μ M) increases the expression of protein substrates phosphorylated by PKC in the presence of PLY (15 ng/ml), which by itself activates this process within 15 min. One of the most important PKC isoforms which is activated in JI-34/PLY-treated cells is PKC-α (Figure 7B, upper panel), since siRNA-mediated PKC-α gene depletion significantly reduces JI-34/PLY-mediated phosphorylation of PKC substrates in these cells (Figure 7B, lower panel). Interestingly, as shown in Figures 8A,B, JI-34 did not further increase LPS (1 μg/ml)-induced PKC-α activation, in contrast to what we observed in PLY-treated cells.

### PKA gene depletion blunts the barrier-protective activity of GHRH agonist in PLY-treated HL-MVEC monolayers

**Figure 9:**
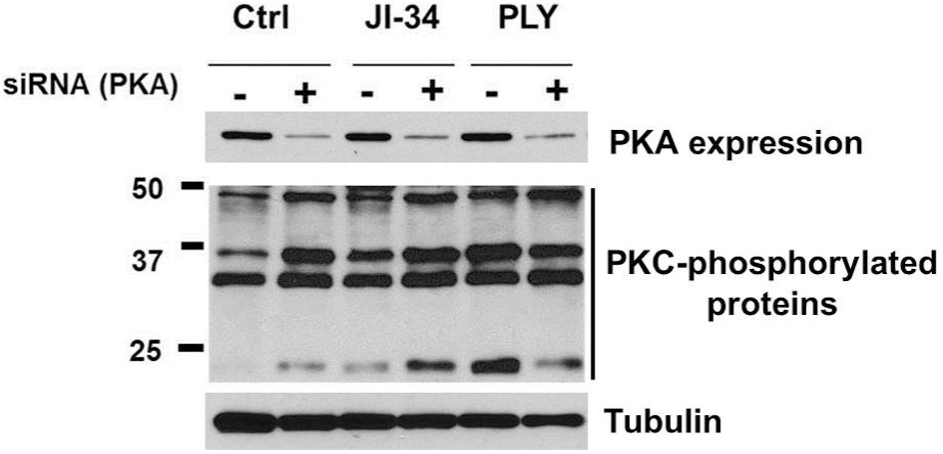
**Representative WB of the influence of PKA-α catalytic subunit siRNA-mediated gene depletion [depicted as siRNA (PKA)] on basal, JI-34- (1 μ M) and PLY- (30 ng/ml) mediated total PKC activation, assessed as phosphorylated PKC substrates**.

In view of our previous observations that JI-34 treatment can increase both barrier-protective PKA activity and barrier-disruptive PKC-α signaling in the presence of PLY, we next investigated the outcome of siRNA-mediated gene depletion of the PKAα catalytic subunit, on the JI-34-induced PKC activation in HL-MVEC monolayers. As shown in Figure 9, PKAα catalytic site (cat) gene depletion significantly increases basal and JI-34-induced PKC activity, measured as the expression of phosphorylated PKC protein substrates, as compared to scrambled siRNA transfection.

**Figure 10:**
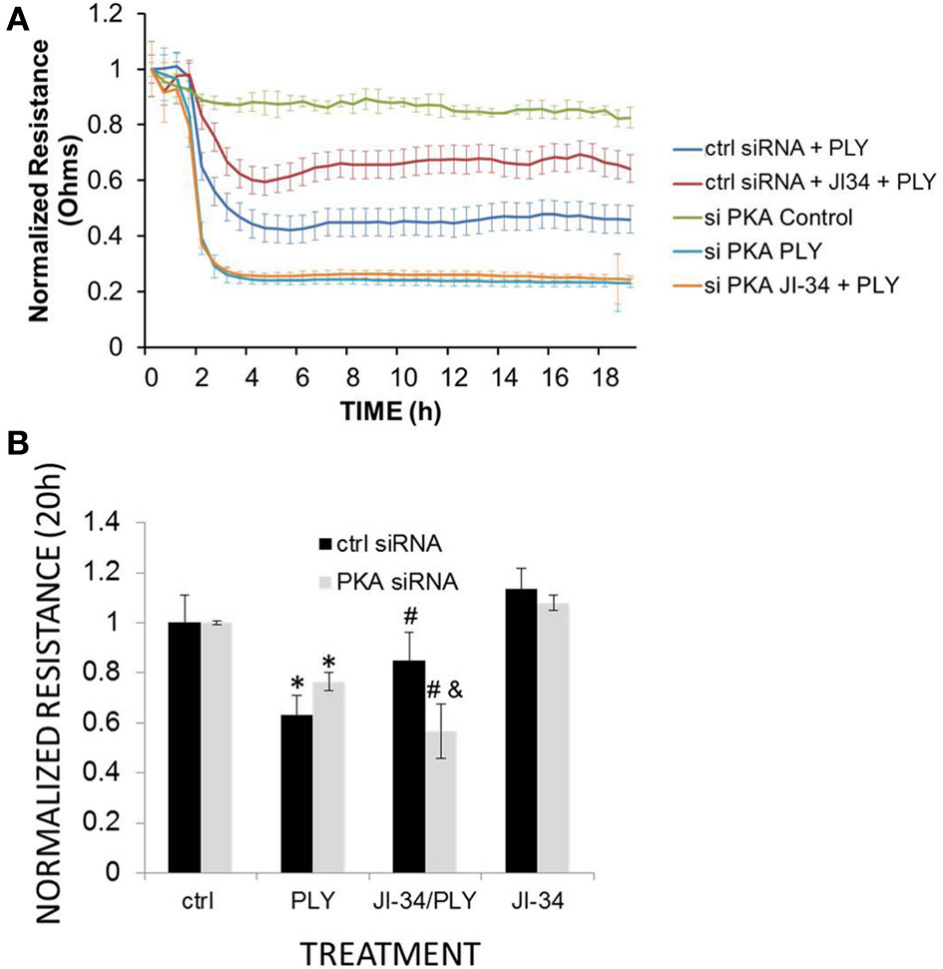
**(A)** TER of HL-MVEC monolayers transfected with either scrambled (control) or PKAα cat. siRNA, that were treated with vehicle, JI-34 (1 μ M), PLY (30 ng/ml), or JI-34 + PLY. *N* = 4 per group and data are depicted as mean ± *SD*. **(B)** TER at 20 h of ctrl or PKA-depleted HL-MVEC treated with JI-34 (1 μ M), PLY (15 ng/ml), or JI-34 + PLY. ^*^p < 0.05 vs. ctrl; ^#^*p* < 0.05 vs. PLY in control siRNA-treated cells; ^#, &^*p* < 0.05 vs. PLY in siRNA (PKA) transfected cells.

As shown in Figure 10A, PKA gene depletion blunts the protective effect of JI-34 toward PLY (30 ng/ml), seen in scrambled siRNA-treated cells. Monolayer resistance of untreated scrambled siRNA-treated HL-MVEC monolayers is not significantly different from untreated control cells (data not shown). PKAα cat siRNA-transfected cells display an increased sensitivity to PLY (Figures 10A,B). The latter result is somewhat in contrast with the equal total PKC activity detected in Western blotting (Figure 9), but could represent an increased activation of barrier-disruptive (such as PKC-α) over barrier-protective PKC isoforms (such as PKC-δ) in HL-MVEC, which would not necessarily be detected in the total PKC activity. As shown in Figure 10B, at a lower PLY concentration (15 ng/ml), the protective effect of JI-34 may shift even to a deleterious one in PKA-gene depleted cells.

## Discussion

Both the GHRH-R and the bioactive SV1 receptor, the latter of which is expressed in HL-MVEC, are coupled to heterotrimeric G proteins (Mayo et al., 1995; Sherwood et al., 2000; Kiaris et al., 2011). The latter consist of α, β, and γ subunits and function as transducers of signals from G-protein coupled receptors (GPCRs). Whereas the α subunit is the GTP binding protein, the β and γ subunits are anchored to the plasma membrane and bind the GDP-bound α subunit with high affinity to constitute the heterotrimer. Although the barrier-protective roles of cAMP and of PKA are well-documented (Bogatcheva et al., 2009), the JI-34-induced interplay between the barrier-protective PKA, induced upon GαS activation and the barrier-disruptive PKC-α, activated by Gαq, represents a unique situation. This can be especially relevant in the presence of bacterial toxins, such as PLY and LPS, which were both shown to activate PKC-α (Witzenrath et al., 2006; Lucas et al., 2012b; Zhao et al., 2013). Thus, unraveling the mechanism by which agonists of GHRH exert their protective effect under these complex circumstances is important. Pathways promoting hyperpermeability in PLY- or LPS-treated MVEC include both MLC-dependent mechanisms and microtubule de-polymerization, the latter of which can cause disassembly of adherens junction proteins, such as VE-cadherin (Petrache et al., 2003; Lucas et al., 2012a). By contrast, activation of PKA, by means of cAMP, was shown to stabilize barrier function in the presence of LPS and PLY (Bogatcheva et al., 2009; Umapathy et al., 2010).

We show in this study that the GHRH agonist JI-34 not only protects HL-MVEC monolayers from PLY-induced barrier dysfunction, but also from LPS-mediated impairment of VE cadherin expression. This adherens junction protein is a relevant parameter of barrier function during acute lung injury. Indeed, a significant reduction of VE cadherin expression was recently detected in lung capillaries of patients with G^−^ sepsis-induced ARDS (Herwig et al., 2013). As such, the VE cadherin expression-restoring activity of JI-34 can provide a plausible explanation for the observed protection from capillary leak in mice which have been instilled i.t. with LPS. Indeed, the improved barrier function in GHRH agonist/LPS-treated animals is not a consequence of a reduced inflammatory response, but rather of an inhibition of direct effects of LPS on the integrity of the lung capillaries. Our data have also shown that of the two pathways induced by cAMP generation following GαS activation, Epac and PKA, in particular the latter seems to be crucial for the protective effect of the GHRH agonist in PLY-treated cells. Indeed, the Epac agonist 2′-O-Me-cAMP failed to protect HL-MVEC monolayers from PLY-mediated barrier dysfunction, in contrast to cAMP (Umapathy et al., 2010) and siRNA-mediated depletion of PKA completely blunted the protective effect of JI-34.

Activation of PKA has been demonstrated to attenuate both epithelial and endothelial barrier dysfunction (Lawrence et al., 2002; Fukuhara et al., 2005). In the endothelium, PKA activation can blunt ROCK activity by direct phosphorylation of RhoGDI and prevention of the release of RhoA from the RhoA-RhoGDI complex (Qiao et al., 2008). Cyclic AMP potentiates VE-cadherin-mediated cell-cell contacts, causing enhanced endothelial barrier function (Dejana et al., 2008). Hence, our observed inhibitory activity of GHRH agonist, JI-34, on PLY- and LPS-mediated VE-cadherin loss can at least partially be explained by its capacity to activate cAMP/PKA pathway, upon binding to the SV1 receptor, expressed in HL-MVEC.

As demonstrated in this study, JI-34 treatment, beyond activating PKA in PLY-treated HL-MVEC, also further increases PLY-induced activation of the PKC-α isoform of PKC. This kinase mediates PLY-induced endothelial hyperpermeability, upon stimulating the barrier disruptive RhoA/ROCK pathway, which increases MLC phosphorylation, actomyosin contractility and intercellular gap formation. Although JI-34 increases PLY-induced PKC-α activity, it does not affect LPS-induced activation of this enzyme. This could potentially be linked to the fact that LPS increases several PKC isoforms, whereas PLY, which induces a large influx of extracellular Ca^2+^ upon pore formation in HL-MVEC, can preferentially activate Ca^2+^-dependent isoforms, of which PKC-α is the main representative.

Although the stimulation of the SV1 receptor in HL-MVEC induces two apparently opposing pathways in the presence of PLY. On the one hand, GHRH agonist stimulates the barrier-protective cAMP/PKA pathway and on the other hand it increases the barrier-disruptive PKC-α/RhoA/ROCK pathway. Our results demonstrate an overriding role for the PKA pathway over the PKC-α pathway under these conditions. However, as shown in Figures 10A,B, when the PKA pathway is impaired, the PKC pathway prevails. Taken together, these results suggest that GHRH agonists will protect from PLY-induced hyperpermeability provided the cAMP/PKA pathway is intact.

Lesions of specific anatomical sites in the brainstem and hypothalamus may initiate acute pulmonary edema. This occurrence has been termed neurogenic pulmonary edema. Unfortunately, the pathophysiologic explanation of this response is not clearly understood. Although hydrostatic derangements may explain certain cases of neurogenic pulmonary edema, recent clinical and experimental studies have indicated that central nervous system disorders may cause a permeability defect without a vascular insult (Colice et al., 1984). The mediating factor for this permeability defect is not clear. It is therefore tempting to hypothesize that peripheral actions of GHRH produced in the hypothalamus may control pulmonary barrier integrity. When this production is impaired, as may occur from brainstem lesions, this may affect pulmonary barrier integrity.

Since GHRH agonists have a longer half-life than the native hypothalamic GHRH hormone (Izdebski et al., 1995; Cai et al., 2014), they can provide a more potent signal to damaged lungs and could promote a change in the concept of treating pulmonary edema. GHRH agonists could improve lung function in pathologies associated with pulmonary permeability edema, such as CNS trauma, ARDS, and severe pneumonia.

### Conflict of interest statement

The authors declare that the research was conducted in the absence of any commercial or financial relationships that could be construed as a potential conflict of interest.
